# The Hidden World Within: Microbial Dynamics in Root Canal Systems

**DOI:** 10.7759/cureus.60577

**Published:** 2024-05-18

**Authors:** Shweta Sedani, Simran Kriplani, Akash Thakare, Aditya Patel

**Affiliations:** 1 Department of Conservative Dentistry and Endodontics, Sharad Pawar Dental College and Hospital, Datta Meghe Institute of Higher Education and Research, Wardha, IND

**Keywords:** bacterial virulence factors, endodontic pathogens, root canal disinfection, bacterial biofilms, microflora

## Abstract

Several hundred different microbial taxa have made the oral cavity their home because of their evolution in multiple species communities within the special ecosystem. On the other hand, the dental pulp or internal tissue of the tooth is a connective tissue that is physiologically sterile and where any microbial infiltration is a harmful indication. It causes the pulp tissue to become inflamed, which leads to the death of the pulp and diffuses infection with inflammation to the peri-radicular tissues. Comprehending the biology of biofilms, the microbial makeup, and the host's reaction to infections in the pathobiology of root canal infections has received a lot of attention throughout the last few decades. Such comprehensive knowledge is required to design preventive medicines as well as clinically effective treatment regimens. Surprisingly, clinical approaches have concentrated more on radiographically perfecting channel preparation than on debridement of these intricate root canal systems, despite the clear realization that root canal infections are biofilm mediated. Since the present comprehension of the microbial etiopathogenesis of apical periodontitis highlights the significance of focusing on procedures such as "canal cleaning" and chemo-mechanical disinfection, the exclusive purpose of endodontic therapy is mainly missed while discussing "canal shaping." We thoroughly examine the state of our knowledge of the composition and functional traits of the root canal microbiome in this review. We also go into the difficulties with root canal disinfection and the cutting-edge approaches that try to solve these difficulties. In conclusion, we present essential guidance for prospective research areas, underscoring their significance as crucial considerations in the field of frontiers in oral health.

## Introduction and background

Despite its obvious importance, the use of microbiology in endodontic therapy has remained debatable for the majority of the 20th century [1]. The calcified walls of the tooth enclose the dental pulp. As a result, even a small amount of dental pulp inflammation can result in pressure that is difficult to release and may eventually induce pulpal necrosis [1]. However, there is one undeniable fact that without microbial involvement in the pulp and associated periapical tissues, there would be no need for endodontic therapy [1]. Kakehaashi et al. who observed that no pathologic alterations occurred in the exposed pulps or peri-radicular tissues of germ-free rats demonstrated this [1]. Conversely, exposure caused peri-radicular lesions and pulpal necrosis in conventional rats. Since mixed microbial infections with the presence of bacteria cause pulp inflammation, abscess, swelling, and discomfort, periapical lesions culture has gained significance [2]. The intricate structure of the mixed infection that frequently underlies clinical situations is better understood from a scientific standpoint thanks to modern anaerobic culture techniques [2]. To comprehend the illness process and determine the best course of therapy, one must be aware of the bacteria linked to endodontic infections [2].

## Review

History

**Figure 1:**
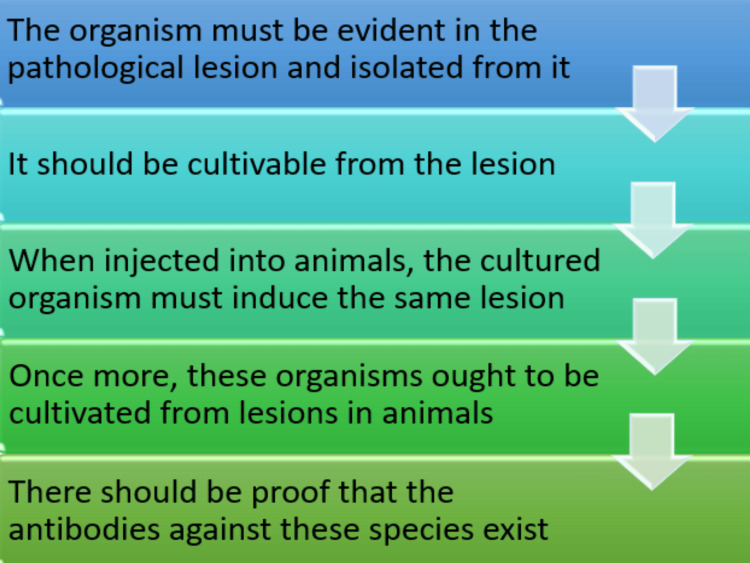
Koch standards of bacterial requirements Image Credits: Dr. Shweta Sedani

An Italian physician Girolamo Fracastorius, was credited with being the first to discover the existence of microscopic living things in 1546. After observing the soft matter of severely damaged teeth's root canals in 1667, Antony van Leeuwenhoek came to the conclusion that these living things resembled the "Animalcules" he had been studying at the time [2]. He has described the different kinds of bacteria. In addition, he created the first microscope [2]. The discovery that fermentation is the outcome of microbial activity was made by Louis Pasteur, the father of microbiology, in 1857. Different kinds of microorganisms were linked to different forms of fermentation [2]. The methods for collecting bacteria in pure culture using solid media were also introduced by Robert Koch, the “Father of Bacteriology” in 1876 [2,3]. Before assigning blame to the organism causing the disease, he proposed some standards. Koch makes the following claims (Figure 1) [3].periodontal ligament

Joseph Lister, the pioneer in antiseptic surgery, sprayed carbolic acid over the wound during a surgery in 1854. The relationship between microorganisms and pulpal and periapical diseases was discovered after nearly 200 years of research and was first noted by Willoughby Dayton Miller, the father of oral microbiology,” in 1890. Working for Robert Koch, Miller created techniques for coloring bacteria in smears and introduced solid media technologies that allowed for the extraction of pure cultures from mixed illnesses. Miller noted that the microorganisms in the pulp chamber of teeth with an open pulp chamber differed from those in root canal therapy. Additionally, he disclosed that a limited number of bacterial strains were viable for cultivation. The study revealed the prevalence of obligatory anaerobic bacteria, constituting 90% of the bacterial flora in infected root canal cultures. In 1936, Fish and McLean demonstrated the absence of microorganisms in the histological examination of the periapical and pulpal tissues of essential and healthy teeth [3]. Naidorf made the following list of generalizations about organisms isolated from root canals (Table 1) [3-5].

**Table 1:** Naidorf list of generalizations of bacteria RCT: root canal treatment

Serial no.	Generalizations
1	The diverse range of organisms identified in root canals is partially influenced by primary cultural techniques [3]
2	The pulpal invasion of dentin has been documented, but there is limited understanding of the types of organisms, their growth rate, and viability [3,4]
3	Pulpal isolates resemble oral flora, with gram-positive cocci being predominant [4]
4	Anaerobes make up 25% of the isolated organisms. Organisms linked to outbreaks are not distinct from those that don't cause symptoms [4]
5	Organism cultures taken from infected canals exhibit various invasive enzymes [4,5]
6	The recommended approach involves addressing the evident origin of infection, i.e., RCT [4,5]

Mac Donald discovered gram-positive organisms in root canals in 1957; streptococci and other bacteria were the most common. Sandquist examined approximately 32 cases for his dissertation, which was published in 1976. He processed and collected microbiological samples using a mobile anaerobic laboratory and an anaerobic glove box. Griffee was the first to compare the signs of particular bacteria and endodontic diseases in 1980. They discovered that the presence of porphyromonas and prevotella species was statistically associated with a higher incidence of symptoms of pain, bad-smelling breath, sinus tract, percussion sensitivity, and localized edema. Yoshida's discovery of peptococcus, porphyromonas, and prevotella from teeth exhibiting clinical symptoms in 1987 supported this [4]. A clear correlation between the progression of an endodontic infection with symptoms and the generation of enzymes by anaerobic bacteria, including species of porphyromonas, eubacterium, prevotella, and peptococcus, was demonstrated by Odell Siqueira et al. in 1996. Collagenase, chondroitinase, and hyaluronidase are these enzymes. Baumgartner (1999) noted that *Prevotella nigrescens* was distinguished from *Prevotella intermedia* by DNA investigations and was the major anaerobe in endodontic infections. Generally believed there are five ecosystems within the oral cavity [4,5]. They are tongue, buccal mucosa, tooth-adherent bacteria coronal to the gingival margin (supra-gingival), bacteria residing apical to the gingival margin (sub-gingival), and saliva (Table 2) [4,5].

**Table 2:** Types of bacteria based on morphology

Type	Description
Cocci	Berry-meaning word, they have a sphere-like shape, they are referred to as streptococci (organized in chains) and staphylococci (clusters similar to a bunch of grapes) [4]
Bacilli	Derived from baculus, meaning rods, they are cylindrical or rod-shaped, corynebacteria have a Chinese letter layout [4]
Vibrio	Curved, comma-shaped rods that get their name from their distinctive vibratory movement, for example, *Vibrio cholerae* [4,5]
Spirochaetes	Named for the coil-shaped speria and the hair-like chaete, they are comparatively longer, slender, flexible creatures with several coils [4,5]
Actinomycetes	Branching filamentous bacteria that are named for their resemblance to sunrays (actis=ray, mykes=fungus) [4,5]

Based on their morphology, bacteria are categorized as follows

Bacterial Anatomy

The two parts of the outermost layer are the cytoplasmic membrane and the stiff cell wall. This contains protoplasm, which is made up of the nuclear body, cytoplasm, and cytoplasmic inclusions such as granules, vacuoles, ribosomes, and mesosomes. The cell could be encased in a viscous layer that is either arranged like a capsule or loose like a coating of slime. Certain bacteria have filamentous appendages that protrude from their cell surface. These include the flagella, which are used for motility, and the fimbriae, which appear to be an attachment organ. Many years have been spent studying the bacterial flora of root canals [4,5]. Previous studies have detailed a flora primarily composed of facultatively anaerobic and aerobic bacteria. Henrics and Hartzell (1919) discovered that S*treptococcus viridans* accounted for 63% of the samples, with *Staphylococcus albur* (17%), *Diptheroid bacilli* (96.5%), and spore-bearing aerobes following closely behind. According to Sommer and associates, hemolytic streptococci and beta-hemolytic streptococci constituted the majority of the organisms isolated from root canals, accounting for only 2% of the total. While Grossman Slack and colleagues discovered identical organisms, they frequently differed in the sample under test. When Grossman cultured 300 consecutive root canals before treatment, he discovered gas-producing organisms in many of them [4,5]. Routes of microbial infiltration or pathways leading to pulpal and periapical infections: microbes are able to enter the dental pulp (Table 3) [4,5].

**Table 3:** Multiple microbial entry routes PDL: periodontal ligament

Serial no.	Entry route
1	From oral cavity [4,5]
2	From the tubules in the dentin [4,5]
3	Through the PDL or gingival sulcus [4,5]
4	By means of anchoress and the bloodstream [4,5]
5	From a damaged occlusal/ill-restored tooth [4,5]
6	By spreading the periapical pathology from neighboring affected teeth [4,5]

Through the open cavity

When intact, enamel and dentin give the pulp further defense against inflammation. But when they sustain trauma or dental cavities, the underlying pulp is invaded. A fresh layer of reparative dentin may be applied as the irritant gets closer to the pulp in order to prevent exposure. Rarely, but occasionally, microbes are kept from accessing the pulp by the quick deposition of reparative dentin. After two weeks, there is less than 2 mm of bacterial penetration. When a healthy vital pulp is exposed due to damage, microorganisms enter the tissues comparatively slowly. Dead tracts of empty dentinal tubules are quickly pierced if the pulp is necrotic [4,5].

Through the dentinal tubules

Since most bacteria have a diameter of less than 1 µm and dentinal tubules have a diameter of 1 to 4 µm, germs can readily pass via the dentinal tubule and reach the pulp. Microorganisms from the preparation's surface may be forced into the pulp by the pressure of cement, impression materials, temporary restoration materials, and dentinal tubules. When cleaning teeth and preparing cavities for complete crowns, this effect needs to be considered. Studies have shown that prepped and open dentin up to a thickness of 0.2 mm can infect the pulp. If caries is the cause of the exposure, treating the caries can help protect the pulp; otherwise, When microbes from the dentinal tubule enter the pulp tissues, the inflammatory reaction enlarges [3,4]. In cases where the dental pulp is infected through the dentinal tubule, the presence is often indicated by specific bacterial strains, primarily facultative anaerobic bacteria like lactobacilli and viridans streptococci, which exhibit a preference for the dentinal tubules. Without direct exposure, pulpitis could result from it. Dentinal tubules are exposed to the oral flora during periodontal therapy when the cementum is removed [4].

Through the gingival sulcus or periodontal ligament

Through the lateral accessory or furcation canals, or through the portals at the apex of the root, microorganisms from the periodontal ligament (PDL) may enter the pulp. According to Langerland et al., pulpal necrosis happens when the apical foramen is impacted. Through the lateral foramina, the canal may be exposed to the bacteria found in the gingival sulcus, if the periodontal disease deteriorates the soft tissue and bone that shields the tooth. In this manner, pulp exposure happens with a high influx of irritants but without cavities or trauma [5].

Through the bloodstream

Anachoresis refers to the movement of microbes through the bloodstream to the inflamed area, leading to infection. While observed in animals, its role in causing significant diseases in humans is not widely acknowledged. It is quite plausible that certain teeth that have been traumatized may get infected by anachoresis. The localization of transitory bacteria in the circulation into an inflammatory region, like traumatized or irritated pulp, is known as anaphoresis [5].

Through an occlusally fractured seal (on a poorly done endodontic therapy-treated tooth restoration)

It has been demonstrated by Torabinajed et al. that in canals sealed with gutta-percha and sealer, salivary contamination from the occlusal aspect can reach the periapical area in less than six weeks [5]. Bacteria may enter the periapical tissue and cause infection if restorative treatment is delayed, in the event that the temporary seal is ruptured, inadequate final restoration as a result of future decay, or if the tooth structure breaks prior to the final repair.

By the spread of an infection from neighboring infected teeth to the periapical region

It is highly uncertain if germs from a periapical region will infiltrate a neighboring tooth that is not diseased. Even though they are the result of pulp necrosis in just one tooth, large periapical radiolucencies can seem to enclose the roots of several teeth. The mandibular anterior teeth are the most commonly affected. When the causing tooth is treated endodontically, the entire radiolucency recovers. Notwithstanding the granulomas, blood vessels and nerves are able to pass through the lesion without risk. A neighboring tooth that has been significantly affected by pulpitis or trauma does have an infected periapical area that requires endodontic treatment as well [5].

Recent taxonomic changes

The black-pigmented bacteria that were formerly found in the genus Bacteroides are now classified as prevotella and porphyromonas genes. Porphyromonas are asaccharolytic. Saccharolytic prevotella is present even in the non-pigmented species. Pathogenicity is the germs' capacity to cause illness. The capacity of a specific strain to cause illness is known as virulence. Generally speaking, filamentous forms, staphylococci, diphtheroids, fusiform bacteria, and other germs are less common than streptococci. According to certain research, gram-negative anaerobic bacteria predominate in endodontic infections. Until the cause of irritation is eliminated through debridement, endodontic disease will continue to exist. To eradicate the bacterium, chemo-mechanical debridement with an appropriate irritant should be carried out. One of the most popular irrigating solutions is sodium hypochlorite (NaOCl) because of its good-tissue dissolving properties and strong antibacterial activity against aerobes, facultative anaerobes, obligate anaerobes, and gram-positive and gram-negative bacteria. Although NaOCl is a useful antibacterial irrigant, larger quantities of it can be harmful to the periapical tissues. When there is at least a 10-minute contact period, NaOCl performs well. In addition to being often used as an irrigating solution or an intracanal medication in between sessions, chlorhexidine has also been recommended for use in the treatment of periodontal disease. Numerous microorganisms, such as gram-positive, gram-negative, bacterial spores, lipophilic viruses, yeasts, and dermatophytes, are susceptible to its action. When present at high concentrations, it is bactericidal and bacteriostatic, respectively. Antibiotic therapy is typically not necessary for pulpitis that is symptomatic. An irritant's removal relieves pain [5].

Microorganisms associated with necrotic pulps

Obligate and facultative anaerobes as well as a fungus have been identified from necrotic pulps; the most common bacteria found there are prevotella, Fusobacterium, lactobacillus, streptococci, clostridium, and peptostreptococcus. A fungus has also been isolated from a few cases [5]. There are several obligate anaerobes, including prevotella, porphyromonas, Veillonella, and Actinomyces, in acute endodontic lesions. *Porphyromonas endodontalis* and *Porphyromonas* *gingivalis* were frequently isolated from teeth exhibiting acute symptoms such as swelling, discomfort, open sinus tract, and percussion sensitivity. Furthermore, *Prevotella denticola* was isolated from teeth that showed no symptoms. The microbiological assessment reveals that the infection may linger even after treatment and is primarily associated with obligatory anaerobes in the case of polymicrobial infections. The most used irrigant is NaOCl, while chlorhexidine gluconate can also be employed due to its broad antimicrobial spectrum. When used in combination with chlorhexidine, NaOCl has superior antibacterial action compared to when used alone. An intracanal medication is not necessary if the endodontic treatment is finished in a single visit; however, if it requires multiple visits, chlorhexidine can be administered as an intracanal medication. Generally speaking, antibiotic coverage is not required unless it is accompanied by systemic symptoms like fever and malaise. The antibacterial efficacy of 2% chlorhexidine gluconate as a root canal irrigant was assessed by Leonardo et al. in 1999 for teeth with radiographically evident chronic periapical lesions and pulpal necrosis. They came to the conclusion that it works just as well in both scenarios. The effects of 1% NaOCl irrigant and 2% chlorhexidine rinse were investigated by Ahmad et al. in 2003. During BMP, they used 1% NaOCl. After that, they used 2% chlorhexidine to flush the canals, dried them, and utilized Ca(OH)₂ as an intracanal medication. Out of the 12 teeth, only one had isolated germs, compared to seven in the control group [6].

Microorganisms associated with teeth with periapical lesions

Until they were linked to an abscess or cellulitis, it was thought that bacteria were primarily restricted to the root canal system of an affected tooth. These infections, however, can involve multiple strains of bacteria. Six patients with asymptomatic periapical lesions were studied by Tronstad et al., who also isolated propionibacterium, *P. gingivalis*, and *P. endodontalis*. An anaerobic infection frequently results in fever, edema, and discomfort. The presence of anaerobic metabolites such as ammonia, indole, urea, and amino acids is indicated by a purulent unpleasant swelling discharge. The majority of black-pigmented bacteria are found in teeth with symptoms. The majority of them are related to sudden periapical abscesses. *P. Gingivalis *and* P. endodontalis* are isolated from symptomatic teeth. In contrast, *P. intermedia* from teeth are both symptomatic and asymptomatic. The most frequent spirochaete found in teeth with cellulitis or endodontic abscesses was* T. Socranski.* Teeth with peri-radicular lesions, particularly those with weeping canals, are typically not treatable in a single visit. An irrigant of choice is NaOCl [6].

The preferred intracanal medication at the moment is calcium hydroxide also called Ca(OH)₂. Some pulp tissue can be dissolved by Ca(OH)₂, which may also help NaOCl dissolve more organic tissue when it comes time for follow-up sessions. The effectiveness of Ca(OH)₂ has been studied in a variety of vehicles: Ca(OH)₂ + water (H₂O), Ca(OH)₂ + CMCP + glycerine, Ca(OH)₂ + saline, Ca(OH)₂ + polyethylene glycol. The zones of inhibition were greater in Ca(OH)₂ pastes with oily carriers than in those with aqueous solutions [6]. Andreas et al. conducted a study examining the combined antimicrobial effects of Ca(OH)₂ and chlorhexidine on common endodontic pathogens, including *Enterococcus faecalis, Fusobacterium nucleatum, Peptostreptococcus micros, P. gingivalis*,* and Streptococcus intermedius* [6,7]. The results indicated that gram-negative bacteria were significantly eliminated by Ca(OH)₂. Moreover, a mixture of Ca(OH)₂ and chlorhexidine eradicated *P. micros* more quickly and *S. intermedius* compared to Ca(OH)₂ alone. The combination reduced the number of viable germs of Enterococcus but did not totally eradicate them. As an intracanal medication, camphorated paramonochlorophenol has also been utilized; however, it only works for the first 24 hours, eliminating roughly 67% of bacteria; in contrast, Ca(OH)₂ takes nearly 24 hours to become active and eliminates approximately 97% of germs after a month. If the infection is progressive or persistent, or if there are systemic signs and symptoms, an antibiotic treatment should be provided along with the proper endodontic therapy. Fever, malaise, cellulitis, trismus that is not explained, and increasing edema are all indicators of systemic involvement and the infection's spread [7]. Since it is effective against a wide variety of facultative and strictly anaerobe bacteria, penicillin VK is still the first antibiotic of choice. However, it is ineffective against bacteria that produce B-lactamases, such as Staphylococcus, and bacteria with black pigments, such as porphyromonas, prevotella, and Fusobacterium species [7,8].

When used against gram-negative bacilli, particularly those that manufacture lactamase, metronidazole is incredibly powerful. However, it is ineffective against aerobic organisms and provides only mediocre protection against several gram-positive anaerobic pathogens (peptostreptococcus and Actinomyces). Penicillin and metronidazole work together to effectively combat both gram-positive and gram-negative, aerobic and anaerobic microbes, including those that generate B-lactamase. Amoxicillin: for seven to 10 days, an oral dosage of 1000 mg should be followed by 500 mg every six hours. Metrodizole: 250 mg should be taken every six hours after a 400 mg loading dosage. The percentage of people allergic to penicillin is about 10%. Erythromycin may be the recommended antibiotic in certain cases. Given that many anaerobes are becoming resistant to erythromycin, even erythromycin should be used in conjunction with metronidazole treatment. Gram-positive facultative and anaerobic bacteria are included in the antibacterial spectrum of erythromycin. Among them are propionibacterium, bifidobacterium, and eubacterium. Actinomyces israeili, peptostreptococcus, and lactobacillus for seven to 10 days, after an oral loading dose of 1000 mg, 500 mg should be taken at six-hour intervals [8].

Clarithromycin or azithromycin

They are better than erythromycin but have a narrower scope than clindamycin. They must be effective against some anaerobic species linked to endodontic infections apart from the microorganisms that are sensitive to erythromycin. They cause fewer gastrointestinal tract (GIT) issues than erythromycin does. For seven to 10 days, without food, 250 mg or 500 mg of clarithromycin may be administered every six to twelve hours. Azithromycin is recommended to be taken either one hour before meals or two hours after meals. The initial dose is 500 mg on the first day, followed by a daily dose of 250 mg. Clindamycin, effective against aerobic gram-positive and a wide range of anaerobes, such as bacteria that produce B-lactamases ought to be administered with a loading dose of 300 mg, followed by 150 mg every six hours for seven to 10 days. It is particularly helpful in situations where B-lactamase antibiotics have proven ineffective, given its good penetration into bone and joints. When paired with clavulanic acid, amoxicillin becomes effective against the majority of aerobic and anaerobic bacteria that produce B-lactamase. Gram-negative aerobic bacteria including Citrobacter, Enterobacter, Serratia, Pseudomonas, and *Escherichia coli* are not inhibited by it. Severe infections need four tablets every six hours [8]. In 2002, Baumgartner conducted research on the antibiotic susceptibility of bacteria linked to abscesses and discovered that the most effective combination was amoxicillin with clavulanic acid [9]. Metronidazole exhibited the highest level of bacterial resistance; however, in the absence of penicillin or amoxicillin, the microorganisms' susceptibility rose from 93% to 98%.

Microorganisms linked with endo-perio lesions

Microorganisms linked to endo-perio lesions are those found in teeth that were non-vital, diseased, and had intact pulp chambers but no obvious way to communicate with the oral environment, such as Actinomycosis species, Bacteroides species, Fusobacterium species, *Campylobacter sputorum*, eubacterium species, peptococcus species, and a few unknown anaerobic facultative microbes can be found through the gingival fissure. Compared to infections of endodontic origin, *P. endodontalis* appears to be far less common in oral infections. Abscess is of periodontal origin in general. Generally speaking, 30% to 58% of periodontal abscesses contain spirochetes. Additionally, studies on teeth with intact crowns and calcified, necrotic pups have demonstrated the presence of CMV and Epstein-Barr virus, and abscesses of endodontic origin contain less than 10% spermatids. When combined, NaOCl, ciprofloxacin, metronidazole, minocycline, and chlorhexidine gluconate have been shown to totally eradicate stringent anaerobe bacteria when employed as intracanal disinfectants. When it comes to teeth that are linked to endodontic-periodontic relationships, root canal cleaning, shaping, and irrigation are insufficient to completely eradicate cultivable bacteria from the system; instead, a hydroxide dressing and additional medication should be administered in between appointments. In 1999, Calt provided an example of how to eliminate Ca(OH)_2_ dressing from the root canal system using ethylene diamine tetra acetic acid (EDTA) and NaOCl [9].

Microorganisms associated with therapy-resistant teeth

When endodontic therapy fails to heal a periapical lesion, *E. faecalis*, *Actinomyces Israeli*, or *Arachina propionica *are typically present. Fungi have also been identified from these teeth in pure cultures (or) in combination with bacteria, with *Candida albicans* being the most often isolated type. When treating teeth that are resistant to therapy, the irrigating solution's concentration and contact time should be increased. Research has demonstrated that when it comes to fighting *E. faecalis*, 2% chlorhexidine was far more successful than 2.5% NaOCl. During the same time frame, it was discovered that 0.12% chlorhexidine had no effect on *E. faecalis*. Zender et al. discovered that Ca(OH)_2_ combined with irrigating solutions (NaOCl and chlorhexidine) had greater tissue dissolving ability and dentin disinfection capability than the Ca(OH)_2_ + saline mixture [10]. Morgana et al. (2004) assessed the antimicrobial activity of 0.2%, 1%, and 2% chlorhexidine and 0.5%, 1%, 2.5%, 4%, and 5.25% of NaOCl [10]. They discovered that all concentrations of NaOCl and 1% and 2% of chlorhexidine eliminated gram-negative bacteria, such as *Staphylococcus aureus* and *C. albicans*, took 15 seconds, while* E. faecalis* took approximately one minute.

Gram stain

Outlined in 1884 by Gramme, it is employed to research how bacteria appear morphologically. All bacteria are divided into two main groups by gram stain as gram-positive groups and gram-negative groups. The principle is that some organisms maintain the color of a basic stain and are not decolorized, for example, Gentian violet (gram-positive organisms). After being treated with a decolorizing agent, the others lose Gentian violet and pick up the counter, for example, Carbol fuchsin (gram-negative organisms).

Gram-positive structure of cell wall

The layer of peptidoglycan in gram-positive bacteria is thicker (15 nm to 25 nm) compared to that of gram-negative (10 nm to 15 nm). There is no periplasmic gap, and the peptidoglycan and cytoplasmic membrane are tightly linked. Specific components of gram-positive are teichoic acid and teichuronic acid, which are soluble polymers containing glycerol polymers of teichoic acid constitute the majority of surface antigens of gram-positive bacteria. The rest of the components are polysaccharides and proteins. Numerous factors can hinder or interfere with the formation of cell walls. The lysozyme enzyme is found in many tissue fluids and lyses microorganisms. It works by severing mucopeptide bonds within cell walls. In a hypertonic solution, lysozyme acts on gram-positive bacteria to generate a protoplast that includes the cytoplasmic membrane and its contents. The outcome of gram-negative bacteria is called spheroplast, which is distinct from protoplast in that part of the cell wall is kept [10,11].

Gram-negative structure of cell wall

A layer of lipoproteins that joins peptidoglycan to the outer membrane. The outer membrane is a bilayer of phospholipids that contains certain proteins. Lipopolysaccharide (LPS) is composed of a polysaccharide bonded to a complex lipid known as lipid A. The endotoxin from gram-negative bacteria is LPS. It is linked to toxicity and serves as a significant surface antigen known as the O antigen. The area between the inner and outer membranes is known as the periplasmic space, and it is home to several significant proteins [11]. Recognized microbial genera and species in infected root canals include gram-positive, gram-negative, aerobic and facultative anaerobic, anaerobic, and types of cocci and rods (Table 4) [11].

**Table 4:** Types of cocci and rods

Cocci	Rods
Streptococcus [11]	Actinomyces [11]
Staphylococcus [11]	Prevotella [11]
Peptostreptococcus [11]	Bacteroides [11]
Veillonella [11]	Fusobacterium [11]

Culture media

Definition

Culture media provides an artificial environment that mimics the natural conditions required for bacterial growth [11].

Indications

To demonstrate the bacteria, in case of persistent symptoms, in medically compromised patients, patients undergoing immunosuppressive therapy, high-risk patients for endocarditis, and patients with heart valve prosthesis as a procedural check antibiotics sensitivity test is needed [11,12]. Classification of media is described in Table 5 [11,12].

**Table 5:** Classification of media based on consistency and properties

According to the consistency	Depending upon properties or functions
Solid media	Simple media nutrient broth–liquid (peptone water) and solid (nutrient agar) [11,12]
Semi-solid media	Enriched media-blood agar, chocolate agar, tetrathionate broth, and selenite F broth [11,12]
Liquid media	Stuart medium for Gonococci, Wilson and Blair medium, MacConkey’s medium, H. sugar media-pentose, and xylose [11,12]

Contents of culture media

McIntosh and Fildes Anaerobic Jar

The anaerobic jar of McIntosh and Fildes is made up of a glass or metal jar with a screw-closing metal lid that can be sealed airtight. The two tubes on the lid serve as an exhaust and an intake for gas, respectively [12,13]. The lid also features two terminals that are capable of being linked to an electrical source. Culture plates with the infection are put inside the jar. The air inside the outlet tube is removed when the vacuum pump is linked to it. The tap at the outlet is closed. Outlet tube and hydrogen gas cylinder are linked. Electric terminals are attached once the jar has been filled with hydrogen to heat the platinized asbestos. This serves as a catalyst to combine leftover oxygen and hydrogen. It guarantees total anaerobiosis. It also bears the possibility of an explosion. In order to confirm that the jar is anaerobic, an indicator should be maintained and reduced methylene blue is employed. Anaerobically colorless, it takes on a blue hue when exposed to O_2_. An anaerobic glove box is a self-contained anaerobic system with circulation of H_2_, N_2_, and CO_2_ as well as the catalytic conversion of any leftover oxygen to water within it. It costs a lot. Complete anaerobic gut flora investigations should be used. Minimization of O_2_ is done by various reducing agents such as 1% glucose, 0.1% thioglycolate, 0.1% ascorbic acid, fresh animal tissue in a broth, for example, rabbit kidneys, and Robertson cooked meat medium [13,14].

Anaerobic culturing: a clinical concept

Clinicians who seek to culture samples acquired from peri-radicular tissue and root canal. Using a luer lock syringe, aspirate the fluid into an anaport vial for the peri-radicular sample [15]. The fluid should be sent to the culture center within four hours. Root canal sample: access c/p, inject chopped-meat glucose broth into the chamber, pump the medium into the root canal with file, and aspirate fluid luer lock syringe [15].

Reversing culture

Nothing more than a bad culture that, after 48 hours, turns positive. Forty-eight hours of incubation proved that 2% of the cultures were negative, according to Grossman's examination of 1000 cases; nevertheless, after 10 days, the cultures turned positive. The care taken during culture, possible leakage between treatments, and the capability of the culture medium to sustain the growth of microorganisms all play a role in culture reversal [15]. In culturing-indirect immunofluorescence, smears of processed pulp samples were reacted with species-specific polyclonal antisera, and resultant conjugates were then tested by immunofluorescence for cross-reaction against a broad selection of gram-positive and gram-negative oral microorganisms with advantages of less time consumption, less expensive for endo applications. Pantera et al. demonstrated the detection of bacteriodes species in the dental pulp [15]. DNA fingerprinting is the most accurate method [15]. Glickman et al. demonstrated the application of genetic code analysis for bacterial identification [15,16]. A technique called PCR that multiplies the genetic code has been useful in bacterial typing but with disadvantages like being time-consuming and expensive [15,16]. Positive and negative associations between various bacteria based on synergism are mentioned below (Table 6) [15,16].

**Table 6:** Positive and negative association in bacteria

Positive association	Negative association
*Fusobacterium nucleatum* with *Peptostreptococcus micros* [15,16]	*Propionibacterium propionicum* against *Veillonella parvula* [15,16]
*Porphyromonas endodontalis, Selenomonas sputigena *with *Wolinella recta* [15,16]	*Capnocytophaga ochracea* against* Veillonella parvula* [15,16]
*Prevotela intermedia* with *Peptostreptococcus micros* [15,16]	Propionibacteria species against Veillonella species [15,16]

Bacterial associations based on synergism

Bacteriocins, local physiological circumstances, bacterial co-aggregation, physical attraction and binding, and nutritional interactions are considered to be the causes of both the positive and negative relationship. Communities are able to adapt to changes in their immediate environment through microbial interactions. As a result, coronal leakage may permit the growth of facultative organisms and salivary infiltration in the coronal portion of the canal. On the other hand, serum from the canal's apical region can encourage the development of proteolytic bacteria at the tip of the root [16]. Bacteria with capsules, whether gram-positive or gram-negative, may be shielded against phagocytosis. Fimbriae during conjugation, pili, can spread from one bacteria to another and exchange DNA for virulence factors, such as antibiotic resistance [16,17]. LPS when released from the outer membrane of gram-negative bacteria is called endotoxin. Studies have demonstrated that the amount of endotoxin in the tooth canals of teeth with symptoms is greater than the amount in the teeth with no symptoms. Enzymes called endotoxin have several biological effects, including the activation of complement and bone resorption. Studies demonstrated that there is a greater concentration of endotoxin in the tooth canals of teeth with symptoms than in those with no symptoms [16,17]. Extra-cellular vesicles, which resemble their parent bacteria in their trilaminar structure, are derived from the outer membrane of gram-negative bacteria. Polyamines are physiologically active substances that control tissue regeneration, growth, and inflammation. Gram-negative bacteria's outer membrane gives rise to vesicles. Since they have the same surface antigens, they can bind antibodies that are intended to destroy the parent organism and lessen the humoral reaction of the host to infection [16,17].

Bacteria isolated from untreated teeth with periapical rarefactions

Bacteroides species have been associated with clinical signs and symptoms. *P. intermedia* was the species most commonly isolated from endodontic infections. Recent studies show that *P. nigrescens* is the most common species isolated from infected root canals and abscesses [17].

Bacterial communities capable of surviving independently in the root canals

Periapical infections were always associated with polymicrobial infections. The bacterial species that can survive independently in the root canal and elicit a periapical response are *E. faecalis, S. aureus, Streptococcus sanguis, Pseudomonas aeruginosa, *and* Bacteroides fragilis*. Recently added bacterial species to the endodontic flora with advances in molecular biology [18]. Species that are found in higher prevalence with molecular methods are *P. endodontalis, P. gingivalis, F. nucleatum, Pseudoramibacter alactolyticus, Propionibacterium propionicum, *Actinomyces*, Slackia exigua, *and *Mogibacterium timidum*. Bacterial survival depends on bacteriocins, local physiological circumstances, bacterial co-aggregation, and physical attraction. The two most prevalent endodontic infections, *F. nucleatum and P. endodontalis*, were never detected using cultivation methods. Forty percent of the infected root canals included the bacteria *T. forsythia, P. gingivalis*, and *T. denticola* known as the "red complex," which are commonly linked to periodontal disorders [18].

Bacteria associated with extra-radicular endodontic infections

A great controversy in endodontics refers to the occurrence of microorganisms establishing extra-radicular infection in the inflamed peri-radicular tissues. Extra-radicular infection can be caused by intra-radicular infection or be independent there. Actinomyces and Pseudo *P. propionicum* have the exclusive feature of surviving in inflamed peri-radicular tissues. The other species frequently found are Fusobacterium, Bacteroides, campylobacter, treponema, eubacterium, and red complex bacteria [18,19].

Viable bacteria in dentinal tubules of teeth with apical periodontitis

Studies conducted in vitro have demonstrated that bacteria can enter the root's dentinal tubules up to 800 mm. Penetration can occur either from the pulpal side or the periodontal side. A significant number of gram-positive rods are found in the dentinal tubules. The most dominant species are Prevotella, Porphyromonas, *F. nucleatum*, peptostreptococcus, and Actinomyces [19].

Bacteria associated with tenacious intra-radicular infection or failed root canal treatment

A peri-radicular lesion's inability to heal during endodontic therapy is frequently caused by bacteria that are still present in the root canal system and have access to the peri-radicular tissues. Research on endodontically treated teeth that need to be retreated has revealed a high frequency of facultative bacteria [19]. Of the microbes, 79.5% consisted of gram-positive species of Staphylococcus, Enterococcus, Enterobacter, Pseudomonas, Stenotrophomonas, Bacillus, Candida species, and sulfur granules were found in 25% of the cases. *A. israeli, Actinomyces viscosus, *and* Actinomyces naeslundii *were identified [19].

Rate of bacterial invasion

According to Nagaoka, the rate of bacterial invasion increased over time, reaching 1.6 µm/day for the first 25 days and 14 µm/day for the last 120 days. When 210 postoperative days had passed, the deepest bacterial invasion measured 3.0 mm. The odontoblastic process and the collagen fibers in the dentinal tubules are two proposed elements that determine the extent of invasion. In vital teeth, it creates a strong physical barrier that prevents bacterial infiltration; however, as processes deteriorate, this barrier becomes less effective in nonvital teeth. One crucial element in the process of bacterial invasion is dentin permeability. It stays constant in nonvital teeth but diminishes with time in vital teeth owing to decreasing tubular diameter. Dentinal fluid movement is a bacterial development into the dentin that may be partially inhibited mechanically by outward fluid flow in the dentinal tubules caused by intrapulpal pressure [20,21].

Enterococcus faecalis

The facultative anaerobic gram-positive coccus *E. faecalis* is closely linked to endodontic infections. Even though *E. faecalis* makes up a minor percentage of the flora in untreated canals, it is a tenacious bacterium that is important in the etiology of chronic peri-radicular lesions that persist after root canal therapy. It can survive in the root canal as a single microbe or as a major part of the flora, and it is frequently found in a high percentage of root canal failures. It can grow in environments with a pH that is extremely alkaline, that is concentrated in salt, that ranges in temperature from 10°C to 45°C, and it can withstand a temperature of 60°C for 30 minutes. *E. faecalis* is found in 40% of cases of initial endodontic infections and 24-77% of cases of persistent endodontic infections [21,22].

Both virulence and survival factors associated with *Enterococcus faecalis*

Absorbs lengthy durations of malnutrition (viable but not cultivable, or VBNC condition); invades dentinal tubules and binds to dentin; modifies the host's answers; suppresses the lymphocytes' activity; and has lipoteichoic acid, pheromones, aggregation material, cytolysin, and lytic enzymes; uses serum as a dietary supplement; opposes intracanal medications, such as Ca(OH)_2_; rivals other cells; creates a biofilm hemolysin, gelatinase, extracellular superoxide; and aggregation substance as a virulence factor. *A. israeli *species associated with peri-radicular inflammation may be resistant to conventional endodontic therapy. But they are sensitive to both NaOCl and Ca(OH)₂ [23].

Endodontic biofilms

Endodontic biofilms help the survival of the bacteria since they are one of the fundamental survival strategies used by bacteria during periods of famine; they have therapeutic significance. Thus, endodontic failures are caused by these biofilms [24]. They provide a safe environment for the exchange of genetic material among the constituent bacterial colonies. They are able to shield the bacteria from the environment [25]. They can trap nutrients for the growth of microbial constituents. Intrinsic resistance to antimicrobial agents, such as endodontic irrigants and intracanal medications, is provided by the biofilm structure [26,27].

Post-treatment sequelae

Even after root canal therapy is finished, bacteria may still exist. For the following reasons, healing may occur even if germs are shown to be present in the root canal. It is possible that there are not enough leftover bacteria to cause inflammation and peri-radicular infection [28]. Because the peri-radicular tissues cannot reach intra-radicular sites, residual bacteria may persist there. A shortage of nutrients or irreversible alterations to the bacterial biofilm ecology could lead to the death of the remaining bacteria [29]. Failure following root canal obturation might be attributed to residual germs present for the following reasons: they are there in sufficient amounts to cause peri-radicular infection; failures may result from residual germs that manage to penetrate the peri-radicular tissue, bacterial remnants that can endure extended periods of low food availability, and modifications in the ecology of bacterial biofilms may lie dormant until the right circumstances arise and they can become active again [30].

## Conclusions

The microbiome associated with the various endodontic infection types has been thoroughly studied, and the infectious etiology of apical periodontitis is a well-established phenomenon. The current gaps in our understanding of the pathophysiology of disease and how it responds to treatment may be filled in with the use of new analytical technologies. To promote additional advancements in the discipline, financial and human resources should be allocated to microbial research. Here are some suggestions for future study directions. Efforts to improve clinical practice and treatment outcomes using the significant basic information now in existence are urgently needed. Is it possible to find microbial signatures that can predict systemic consequences like diabetes mellitus or cardiovascular disorders, given the growing body of evidence linking endodontic infections to these conditions? Finally, future research may focus on proactive anti-biofilm strategies in addition to microbial impetus targeting to counteract the root canal infection etiopathogenesis mediated by biofilms. These strategies may include inhibition of quorum sensing, dispersion of extracellular polymeric substance, the disintegration of biofilms, and suppression of signaling pathways and macromolecule synthesis. If successful, these approaches could lead to a range of cutting-edge endodontic cleaning techniques. This could open the door for the creation of endodontic disinfection treatments that are more comprehensive and possibly more effective. By addressing these unanswered issues, we may be able to develop a framework for bettering therapy regimens, diagnostic processes, and the likelihood of unfavorable clinical evolutions.
